# Functional Aromatic Poly(1,3,4-Oxadiazole-Ether)s with Benzimidazole Pendants: Synthesis, Thermal and Dielectric Studies

**DOI:** 10.1155/2014/790702

**Published:** 2014-10-08

**Authors:** Shimoga D. Ganesh, Vasantakumar K. Pai, Mahadevappa Y. Kariduraganavar, Madhu B. Jayanna

**Affiliations:** ^1^Department of Industrial Chemistry, School of Chemical Sciences, Jnana Sahyadri, Kuvempu University, Shankaraghatta, Shimoga, Karnataka 577 451, India; ^2^Department of Chemistry and Center of Excellence in Polymer Science, Karnatak University, Dharwad 580 003, India; ^3^Department of Physics, Government Science College, Chitradurga, Karnataka 577501, India

## Abstract

Poly(1,3,4-oxadiazole-ether) with reactive carboxylic acid pendants was synthesized from solution polymerization via nucleophilic displacement polycondensation among 2,5-bis(4-fluorophenyl)-1,3,4-oxadiazole (BFPOx) and 4,4′-bis(4-hydroxyphenyl) valeric acid (BHPA). Without altering the polymeric segments, benzimidazole modified poly(1,3,4-oxadiazole-ether)s were prepared by varying stoichiometric ratios of 1,2-phenylenediamine. The molecular structural characterization of these polymers was achieved by, FT-IR, NMR, TGA, elemental analysis, and analytical techniques. The weight-average molecular weight of virgin polymer with carboxylic acid functionality was determined by gel permeation chromatography (GPC) and was found to be 22400 (Mw/Mn = 2.07). All the synthesized polyethers were compressed into pellets and electrical contacts were established to perform dielectric properties.

## 1. Introduction

High performance polymers are of outstanding significance as light emitting diodes, nonlinear optical devices, and photovoltaic cells [1, 2]. Among these, polymers with heterocyclic groups, for instance, 1,3,4-oxadiazole, are very willfully studied for pronouncing electroluminescent properties [3–5] and to be used in electrochemical sensors [6, 7]. Poly(arylene-oxadiazole)s, which are of special interest for the production of advanced materials due to their high thermal oxidative stability and specific properties, are determined by the electron withdrawing nature of oxadiazole units. Aromatic polyoxadiazoles are known for their excellent thermal resistance and good hydrolytic stability; they have rigid molecules due to the delocalization of *π*-electrons, which makes them insoluble in organic solvents and infusible, and therefore their processing and practical use is very limited [8]. The most part of the recent research aims to obtain structurally modified poly(1,3,4-oxadiazole)s with flexible ether and sulfone linkages for good solubility in organic solvents and lower glass transition temperature, in order to allow their processing from solution, without affecting the properties that give them the status of high performance polymers; such modifications are the insertion of mesogenic substituents on aromatic rings [9], flexible bridges in the macromolecular chains, aliphatic pendants to the chain, and, more recently, the heterocyclic rings as part of the side chain of poly(1,3,4-oxadiazole-ether) [10–13].

Benzimidazoles can be easily constructed via classical synthetic route from the condensations of 1,2-phenylenediamine with carboxylic acid functional group. Indeed, polymers bearing the carboxylic acid functionality can be converted practically to benzimidazoles; polymers with various heterocyclic units are widely used and studied for their good dielectric and pyroelectric performances [14, 15]. Benzimidazole and 1,3,4,-oxadiazole units in main and side chain of the polymer can withstand extreme conditions without an extensive loss of properties and can be widely used in the aerospace industry, where thermal stability is a prime obligation [16]. Both heterocycles are best recognized for their admirable thermal resistance with good electrical properties [17, 18], due to their customary applications; various functional groups [19–22] have paid much attention to the physicochemical properties of polymeric stuff, mainly because of the unique properties of each individual. A key advantage of merging the properties of these two functional units is potential to exhibit collective thermal and dielectric properties.

**Figure 1:**
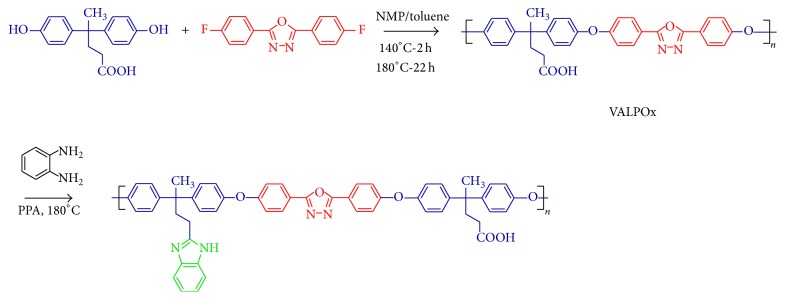
Synthetic route to prepare benzimidazole modified poly(1,3,4-oxadiazole-ether) via VALPOx polymer.

The present paper describes the synthesis of a poly(1,3,4-oxadiazole-ether) that comprises carboxylic acid moiety (Figure 1) and its respective benzimidazole derivatives, afforded by polyphosphoric acid condensation with *o*-phenylene diamine at different mass percentages with respect to the virgin polymer. The monomer and synthesized polymers were well characterized with various spectroscopic techniques, and the dielectric performance of the polymers was investigated.

## 2. Experimental

### 2.1. Materials

4,4′-Bis(4-hydroxyphenyl) valeric acid and 4-fluoro benzoic acid were purchased from Sigma-Aldrich and used as received. *N*-methyl pyrrolidinone (NMP) was purchased from Merckand purified by distillation over phosphorous pentoxide under reduced pressure. Potassium carbonate and 1,2-phenylenediamine (used after recrystallization from hot water) were purchased from HIMEDIA Chemicals. Polyphosphoric acid was purchased from Spectrochem Pvt. Ltd.; all other solvents and reagents are purchased from S.D. Fine Chem. Ltd., Mumbai, India, were of analytical grade, and were used without further purification. Double distilled water was used throughout the study.

### 2.2. Instrumentation

Elemental analyses were performed with a PerkinElmer PE 2400 CHN elemental analyzer. The Fourier transform infrared (FT-IR) spectra were recorded by a Thermo Nicolet −5700, USA, spectrometer (diamond window method). The ^1^H & ^13^C NMR spectra were recorded on a Bruker Avance 400 MHz spectrometer using tetramethylsilane (TMS) as an internal standard reference. The solid-state ^13^C-NMR spectra were recorded on a Bruker DSX-500 solid-state NMR spectrometer with a magnetic field of 7.04 T and carbon frequency of 125.77 MHz (internal standard was glycine). Thermogravimetric analysis (TGA) was performed on a PerkinElmer Diamond TGA/DTA thermogravimetric analyzer at a heating rate of 10°C/min in a nitrogen atmosphere. The number- and weight-average molecular weights (*M*_*n*_ and *M*_*w*_) of VALPOx polymer were estimated by gel permeation chromatography (HP/GPC, Waters). Degassed tetrahydrofuran (THF) was used as eluent at a flow rate of 1.0 mL/min. A set of monodisperse polystyrene standards covering the range of 10^3^–10^7^ was used for the molecular weight calibration.

## 3. Synthesis

### 3.1. Synthesis of the Monomer 2,5-Bis(4-fluorophenyl)-1,3,4-oxadiazole (BFPOx)

Method was adapted from the literature [23]. To a pulverized mixture of 4-fluorobenzoic acid (14.05 g, 10 mmol) and hydrazine sulphate (6.574 g, 5 mmol), 125 g of polyphosphoric acid was added under a moisture free nitrogen atmosphere, into a round-bottom flask equipped with a magnetic stirrer and a condenser. The reaction system was then evacuated and filled with nitrogen for three times to remove air and moisture. The solution was heated to 150°C with stirring under the protection of moisture free nitrogen atmosphere and maintained at this temperature for 6 h, and then the reaction temperature was slowly increased to 200°C over 3 h to affect the ring closure. The solution was allowed to further react for an additional 1 h at this temperature until no more gas release was observed. The reaction mass was then cooled to about 60°C and poured into 500 mL of ice cold distilled water with stirring. The white fibrous material obtained was collected by filtration and washed with saturated sodium bicarbonate solution (to remove unreacted 4-fluorobenzoic acid) and finally washed several times with hot water until the filtrate was neutral. The purification of the crude product was done by recrystallization from a 95% ethanol/THF (90/10) mixture twice, which generated 10.8 g of the pure white crystals with a yield of 85%. MP: 203.2–203.8°C. ^19^F NMR (*δ*, DMSO-*d*_6_): −107.28 (s, 2F, para to Ox). ^1^H NMR (*δ*, DMSO-*d*_6_): 7.43–7.48 (m, 4H), 8.15–8.18 (m, 4H), ^13^C NMR (*δ*, DMSO-*d*_6_): 165.38 (F attached to C), 163.28 (2C, Ox), 162.90 (F attached to C), 129.42 & 129.33 (2C, attached to oxadiazole), 119.97 & 119.94 (4C, ortho to Ox), 116.76 & 116.51 (4C, meta to Ox).

MS: 259.0 (MH^+^); calcd for C_14_H_8_F_2_N_2_O, 258.36. Anal. Calcd: C, 65.12; H, 3.12; F, 14.7; N, 10.85; O, 6.20. Found: C, 65.04; H, 3.01; N, 10.68. FT-IR (diamond window): 1652 (C=N of Ox ring), 1560, 1504, 1422, 1364, 1320, 1214, 1104, 1038, 992, 845, 821, 779, 749, 709 cm^−1^.

### 3.2. Synthesis of (VALPOx) Poly(1,3,4-oxadiazole-ether)

The synthesis of VALPOx with active free carboxylic acid groups was achieved from the nucleophilic displacement reaction of oxadiazole-activated bis(fluoride) monomer with 4,4′-bis(4-hydroxyphenyl) valeric acid.

A typical synthesis of this polymer was conducted in a three-neck flask equipped with a nitrogen inlet, stirrer, Dean-Stark trap, and condenser.

The flask was charged with 2,5-bis(4-fluorophenyl)-1,3,4-oxadiazole, 0.2582 g (1 mmol), 0.2684 g (1 mmol), K_2_CO_3_ (0.2903 g, 2.1 mmol), N-Methyl pyrollidine (10 mL), and toluene (10 mL); the flask was purged with moisture free nitrogen three times. The reaction mixture was then heated to 120°C for 2 h until the toluene was all condensed in the Dean-Stark trap. Upon dehydration, the polymerization was heated to 180°C for 22 h. The cooled viscous reaction mixture was diluted with 5 mL of NMP and then dropped into 300 mL of demineralised water containing 10% hydrochloric acid. The precipitated polymer was repeatedly washed with excess of water and dried in vacuum at 60°C.

Yield: 85%; FT-IR (diamond window, cm^−1^) 1708 (C=O, sym.), 1585 (C=O, asym.), 1504 (–C=N, sym.), 1488, 1243 (–C–O–C), 1070 (C–O, oxadiazole ring). ^1^H NMR (400 MHz, DMSO-*d*_6_): *δ* = 1.44–1.60 (m, 3H), 1.97–2.02 (m, 2H), 2.27–2.37 (d, 2H), 6.63–6.69 (br s, 1H), 6.91–7.05 (br s, 4H), 7.15 (br s, 4H), 7.02–7.22 (m, 1H), 7.26 (br s, 2H), 7.45 (m, 1H), 8.06–8.08 (br s, 3H), 8.16–8.19 (m, 1H) 12.05 (br s, 1H). ^13^C NMR (125 MHz, solid state): *δ* = 30.42, 35.78, 45.13, 121.13, 129.53, 134.37, 143.00, 144.21, 151.74, 162.90, 171.27, 176.52, 177.46.

### 3.3. Synthesis of Benzimidazole Modified VALPOx Polymer

A direct synthesis of a modified VALPOx polyether with 30 mass % 1,2-phenylenediamine (VALPOx-B-30) is discussed. In a 100 mL, three-neck flask equipped with a mechanical stirrer and a nitrogen inlet/outlet, finely powdered 1.0 g (9.0 mmol) of VALPOx and 0.3 g of 1,2-phenylenediamine were added and stirred by adding 20 g of polyphosphoric acid. The reaction system was then evacuated and filled with nitrogen for three times to remove air and moisture. The reaction mixture was stirred at 120°C for 1 h until complete dissolution of the polymer and 1,2-phenylenediamine; then temperature was slowly raised to 150°C for a period of 12 h. The reaction mixture was cooled to about 60–70°C and poured in to 500 mL ice cold distilled water with proper stirring, The dark pinkish brown fibrous material separated was collected by filtration and washed several times with water and finally washed with hot water until the filtrate was neutral. The resulting dark brown polymer VALPOx-B-30 was dried under vacuum at 60°C for 24 h.

Yield: 80%; FT-IR (diamond window, cm^−1^) 3394 (N–H), 1600 (–C=N, sym), 1488, 1243 (–C–O–C), 1069 (C–O, oxadiazole ring). ^13^C NMR (125 MHz, solid state): *δ* = 62.89, 65.25, 71.60, 72.52, 74.93, 84.47, 88.99, 104.37, 118.87, 128.98, 153.54, 156.89, 163.21.

A similar procedure was followed for the synthesis of polymers VALPOx-B-10 and VALPOx-B-20. For example,VALPOx-B-10 describes the VALPOx polymer that incorporates 10 mass % of 1,2-phenylenediamine.

## 4. Results and Discussion

### 4.1. Synthesis and Characterization

The synthesis of new polymer VALPOx with active carboxylic acid moiety was achieved via conventional aromatic nucleophilic substitution polymerization technique from 4,4′-bis(4-hydroxyphenyl) valeric acid and 2,5-bis(4-fluorophenyl)-1,3,4-oxadiazole; the polycondensation was carried out at elevated temperature in NMP/toluene azeotrope in presence of anhydrous pulverised potassium carbonate as catalyst. The carboxylic acid in the virgin polymer is successfully converted to benzimidazole pendants via polyphosphoric acid condensation route. The amount of 1,2-phenylenediamine with respect to VALPOx varied as 10, 20, and 30 mass %, and the polymers thus obtained were designated as VALPOx-B-10, VALPOx-B-20, and VALPOx-B-30, respectively. The incorporation of benzimidazole moiety in polymer was confirmed via FTIR spectroscopy (diamond window method) in the range of 400–4000 cm^−1^; in each scan, the amount of well-grounded sample was kept constant (1 mg) in order to estimate the changes in the intensities of the characteristic peaks with respect to the amount of 1,2-phenylenediamine; the peak at 1070 cm^−1^ was assigned to C–O stretching of oxadiazole and benzimidazole modified poly(1,3,4-oxadiazole-ether) shows 3394 cm^−1^ peak for N–H stretching; NMR spectroscopic studies of synthesized polymers are in agreement with the proposed structure. Signal broadening of the ^1^H NMR spectrum of the polymer VALPOxwas due to polymerization and is presented in Supplementary Material (See Supplementary Material available online at http://dx.doi.org/10.1155/2014/790702). The signals resonated at 12.05 ppm were assigned to carboxylic acid protons, and signals at the range of 2.00 and 2.34 ppm were assigned to methylene protons, indicating the 4,4′-bis(4-hydroxyphenyl) valeric acid moiety introduced successfully, indicating the formation of polyether (VALPOx) with carboxylic acid pendants; further signal at 174.50 *δ* in ^13^C NMR spectra (see Supplementary Material) confirms the carboxylic acid carbon and two methylene carbons at 29.87 *δ* and 36.15 *δ*. The insertion of oxadiazole unit is revealed by the signal at 165.40 *δ*, indicating the oxadiazole unit carbons attached to phenyl rings in polymer chain. Due to insolubility of benzimidazole substituted polymers, solid-state ^13^C NMR was recorded in comparison to VALPOx polymer which was presented in Supplementary Material. It gave sufficient information for successful incorporation of pendant benzimidazole into the polymer. Solid-state ^13^C NMR of VALPOx exhibits broad peak around 176.5–177.4 ppm which corresponds to the carbonyl carbon of the carboxylic acid; peaks at 162.9–172.9 ppm were assigned to carbons of oxadiazole ring; a broad signal at 121.1–134.3 ppm and 30.4–45.1 ppm corresponds to aromatic and aliphatic regions of the polymer chain, respectively; aromatic carbons attached to the oxadiazole unit was observed at 143.0–151.7 ppm. The deshielded aliphatic signals and absence of peak around 176.5–177.4 ppm suggest the successful complete conversion of pendant carboxylic acid to benzimidazole at maximum stoichiometric ratio (VALPOx-B-30). The molecular weight of VALPOx polymer was determined by GPC with polystyrene as the standard and THF as the eluent, showed *M*_*w*_ of 22400 with polydispersity 2.07. The VALPOx polymer was soluble in THF, DMAc, DMSO, and NMP at ambient temperature but the solubility of benzimidazole modified polymers was poor in aforementioned solvents as benzimidazole content upsurges in the polymer. These modified poly(1,3,4-oxadiazole-ether)s were compressed into pellets for further AC electrical studies.

### 4.2. Thermal Analysis

**Figure 2:**
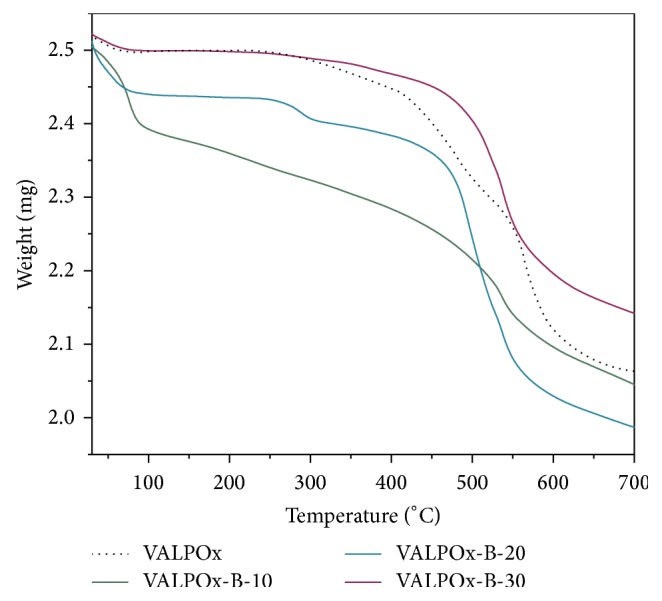
TGA plot of poly(1,3,4-oxadiazole-ether)s at a heating rate of 10°C/min under a nitrogen atmosphere.

Thermogravimetric analyses (TGAs) were performed for all the polymers and the results were presented in Figure 2. Poly(1,3,4-oxadiazole-ether) containing carboxylic acid showed a high-temperature stability with an onset of decomposition at 250°C. Therefore, polymer was modified in bulk at 150°C under nitrogen to acquire the benzimidazole bearing poly(1,3,4-oxadiazole-ether)s. These polymers showed high-temperature stability up to 610°C, due to 1,3,4-oxadiazole unit in the main chain with benzimidazole side groups. The moisture loss is observed before 150°C. Another major steep weight loss range of 240–320°C was noticed in VALPOx, VALPOx-B-10, and VALPOx-B-20 and was assigned to the decomposition carboxylic acid groups from polymer chain, and also we cannot neglect the ionic interactions of carboxylic acid groups with the benzimidazole moieties. The main polymeric backbone degradation step in case of benzimidazole modified VALPOx polymers can be accounted and is in the range of 420–610°C, while VALPOx polymer shows degradations stability in the range 350–640°C owing to the presence of carboxylic acid groups.

**Figure 3:**
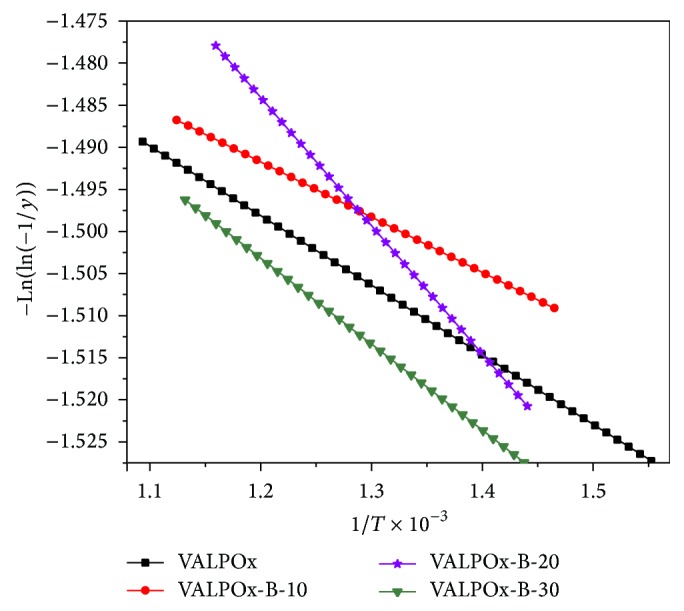
Plots of −Ln(ln(−1/*y*)) versus 1/*T* × 10^−3^ for the decomposition step in the range 350–640°C of VALPOx, VALPOx-B-10, VALPOx-B-20, and VALPOx-B-30 polymer sample.

Kinetic and thermodynamic parameters were determined using Broido's method [24] and tabulated in Table 1. Plots of −Ln(ln(−1/*Y*)) versus 1/*T* (where *Y* is the fraction of the compound undecomposed) were developed for the decomposition segment. From the plot (Figure 3) the activation energy (*E*_*a*_) and frequency factor (ln *A*) were evaluated. The enthalpy (Δ*H*), entropy (Δ*S*), and free energy (Δ*G*) have been calculated.

### 4.3. AC Electrical Measurements

Finely powdered polymer samples were compressed into pellets of thickness in the range of 0.5-0.6 mm and subjected to the dielectric measurements; Sandwiched polymer samples between two silver-plated stainless steel electrodes were analysed by impedance analyzer model HIOKI 3352-50 HiTESTER Version 2.3. Silver paint (ELTECKS preparation number 1228-C) was coated on both of the flat exteriors of the pressed tablet and the electrical contacts were made using the same silver paint to the silver electrodes. The electrical contacts were checked to verify the ohmic connection. The measurements were carried out at room temperature in between the 50 Hz–5 MHz. The capacitance value (*C*) and ac conductance (*G*) were directly obtained from the apparatus. The dielectric constant (*ɛ*′) and ac conductivity (*σ*_ac_) values are calculated using (1) and (2), respectively:

(1) $$\begin{array}{c} {ɛ}^{\prime }=\frac{C_{P}d}{{ɛ}_{o}A}, \end{array}$$

(2) $$\begin{array}{c} \sigma_{\text{ac}}=\frac{Gd}{A}, \end{array}$$

where “*d*” is the thickness of the polymer pellet and “*A*” is the cross-section area and *ɛ*_*o*_ is the permittivity of the free space. All these measurements were performed under dynamic vacuum.

### 4.4. Frequency Dependence of Dielectric Constant (*ɛ*′) and Dielectric Loss (tan*δ*)

**Figure 4:**
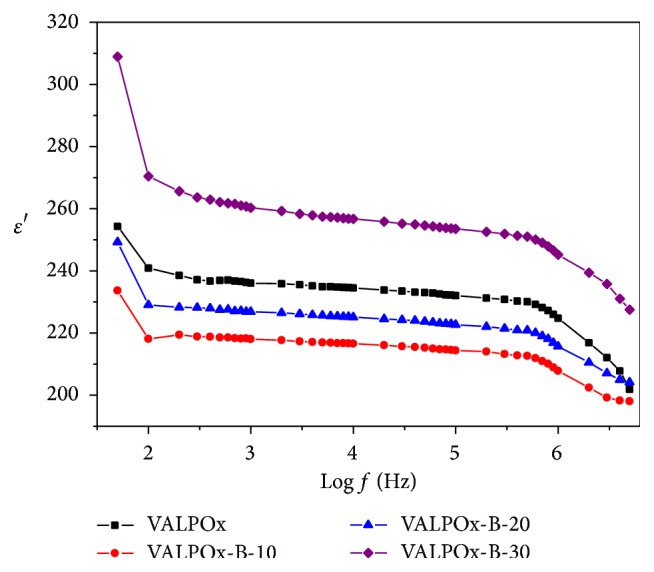
Room temperature variation of dielectric constant (*ɛ*′) with log (frequency) of VALPOx and benzimidazole modified VALPOx polymers.

The variation of dielectric constant versus frequency at room temperature was plotted in Figure 4. The typical decrease in the *ε*′ is observed as polyethers generally follow [25]; at lower frequencies (<100 Hz) there is an increasing contribution to the dielectric constant; this is because at very low frequencies dipoles follow the field. The dielectric constant remains fairly constant at low frequency region; such behavior is attributed to presence of frozen dipoles slowly attaining freedom of rotation at higher frequencies; the charge carriers may migrate via dielectric and get trapped against a defect site; they induce opposite charge in their vicinity; as a result of this motion of charge carriers slowed down and decrease in the *ɛ*′ is observed [26]. Minor upsurge in the *ɛ*′ is witnessed more by VALPOx-B-30 than the virgin VALPOx polymer, owing to the maximal benzimidazole moieties in the polymer segments.

**Figure 5:**
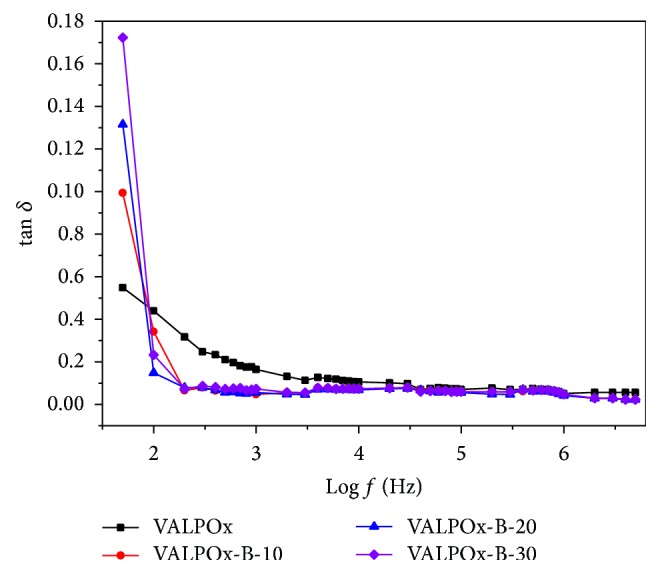
Room temperature variation of loss tangent on log (frequency) for VALPOx and benzimidazole modified VALPOx polymers.

Plot of loss factor (tan*δ*) versus log (frequency) is shown in Figure 5. In accordance with the plot, dielectric loss (tan*δ*) decreases exponentially with increase of frequency up to 4 kHz and attains the constant value due to interfacial polarization. This results supports the view that frequency dependence dielectric loss could be explained using near constant loss (NCL) model [27, 28].

### 4.5. Frequency Dependence of ac Conductivity (*σ*_*ac*_)

**Figure 6:**
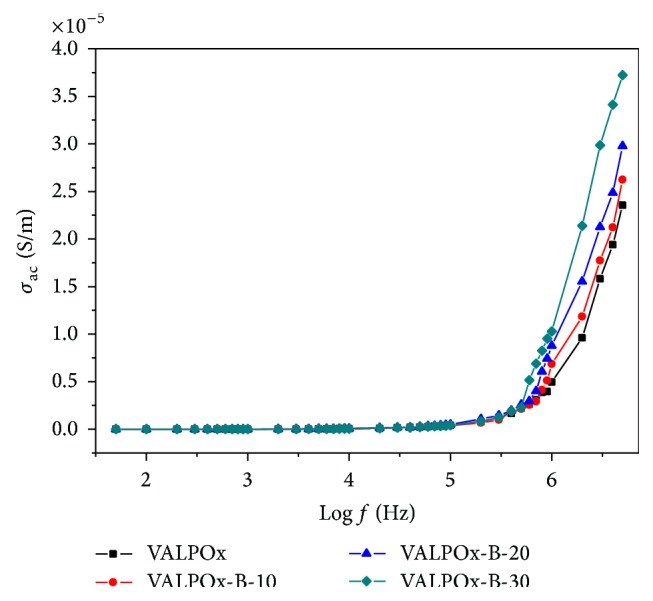
Room temperature variation of AC conductivity with log (frequency) for VALPOx and benzimidazole modified VALPOx polymers.

The frequency dependence of the ac conductivity for VALPOx and benzimidazole modified VALPOx polymers are shown in Figure 6. The plot reveals that conductivity increases with increase of frequency as well as benzimidazole content in the VALPOx; this is due to frequent charge motion within the sample. As the polymer is modified by the benzimidazole moiety, increase in the *σ*_ac_ is noticed; increased benzimidazole units are expected to interact with 1,3,4-oxadiazole segments in the polymeric chain; benzimidazole modification converts the parent polymer structure into free hydrogen depleted carbon network, and as a result ac field of sufficiently higher frequency may cause the net polarization of the polymer, which will make the net polarization in a given field larger and hence the dielectric constant will be higher. which is out of phase with the field. This results in increase of conductivity at higher frequencies.

## 5. Conclusion

Poly(1,3,4-oxadiazole-ether)s with pendant benzimidazole units were synthesized and characterized; it was demonstrated that good thermally stable polymeric stuff can be synthesized from the pendant carboxylic acid functional poly(1,3,4-oxadiazole-ether) (VALPOx); reactive pendant carboxylic acid group is exploited to hold the benzimidazole moieties, by polyphosphoric acid condensation. These polymers with benzimidazole and oxadiazole heterocycles exhibit remarkable thermal stability with decomposition temperature above 410°C. Frequency dependence ac conductivity has been found to support the nearly constant loss (NCL) model in isothermal condition at room temperature. The dielectric constant *ε*′ and dielectric loss (tan*δ*) depend on frequency up to 4 kHz and are nearly constant beyond. In summary, these polymers with significant properties have been demonstrated to be a robust yet flexible class of materials with potential applications in microelectronics as sensory materials and high operating frequency devices.

## Supplementary Material

Nuclear Magnetic Resonance spectroscopic data plots (^1^H, ^13^C and Solid state ^13^C) were provided; it clearly projects the assignments of respective peaks in some detail.

## Figures and Tables

**Table 1:** Kinetic and thermodynamic parameters of polyethers.

Samples∗	Decomposition range (°C)	*E*_*a*_ (kJ/mol) ×10^−3^	ln⁡*A*	Δ*H* (kJ/mol)	Δ*S* kJ/K	Δ*G* kJ/mol
P	350–640	1.582	−9.93	−6.39285	−161.624	124.3015
P-B-10	410–610	1.256	−10.17	−6.52982	−161.222	126.6415
P-B-20	420–590	2.919	−9.18	−6.46377	−161.298	125.4526
P-B-30	422–610	1.953	−9.68	−6.56409	−161.563	125.6582

^*^P = VALPOx, P-B-10 = VALPOx-B-10, P-B-20 = VALPOx-B-20, and P-B-30 = VALPOx-B-30.
