# Supplementary material for: Functional Aromatic Poly(1,3,4-Oxadiazole-Ether)s with Benzimidazole Pendants: Synthesis, Thermal and Dielectric Studies
Source: Int Sch Res Notices. 2014 Oct 8;2014:790702. doi: 10.1155/2014/790702 (PMC4897065; doi:10.1155/2014/790702)
Supplement: Supplementary file 1 — Nuclear Magnetic Resonance spectroscopic data plots (1H, 13C and Solid state 13C) were provided; it clearly projects the assignments of respective peaks in some detail. [file 790702.f1.pdf]

**SUPPORTING INFORMATION**

# Functional Aromatic Poly(1,3,4-oxadiazole-ether)s with benzimidazole pendants: Synthesis, thermal and dielectric studies

**Shimoga D Ganesh[1], Vasantakumar K Pai [1,] *, Mahadevappa Y Karidurganavar[2], Madhu B Jayanna[3],**

[1]Department of Industrial Chemistry, School of Chemical Sciences, Jnana Sahyadri, Kuvempu University, Shankaraghatta - 577 451, Shimoga, Karnataka, INDIA

[2]Department of Chemistry and Center of Excellence in Polymer Science, Karnatak University, Dharwad - 580 003, INDIA

[3]Department of Physics, Government Science College, Chitradurga-577501, Karnataka, INDIA

*Author for correspondence (vasantapai@gmail.com)

Tel.: +91-08282-256228(O); Fax: +91-08282-256255,

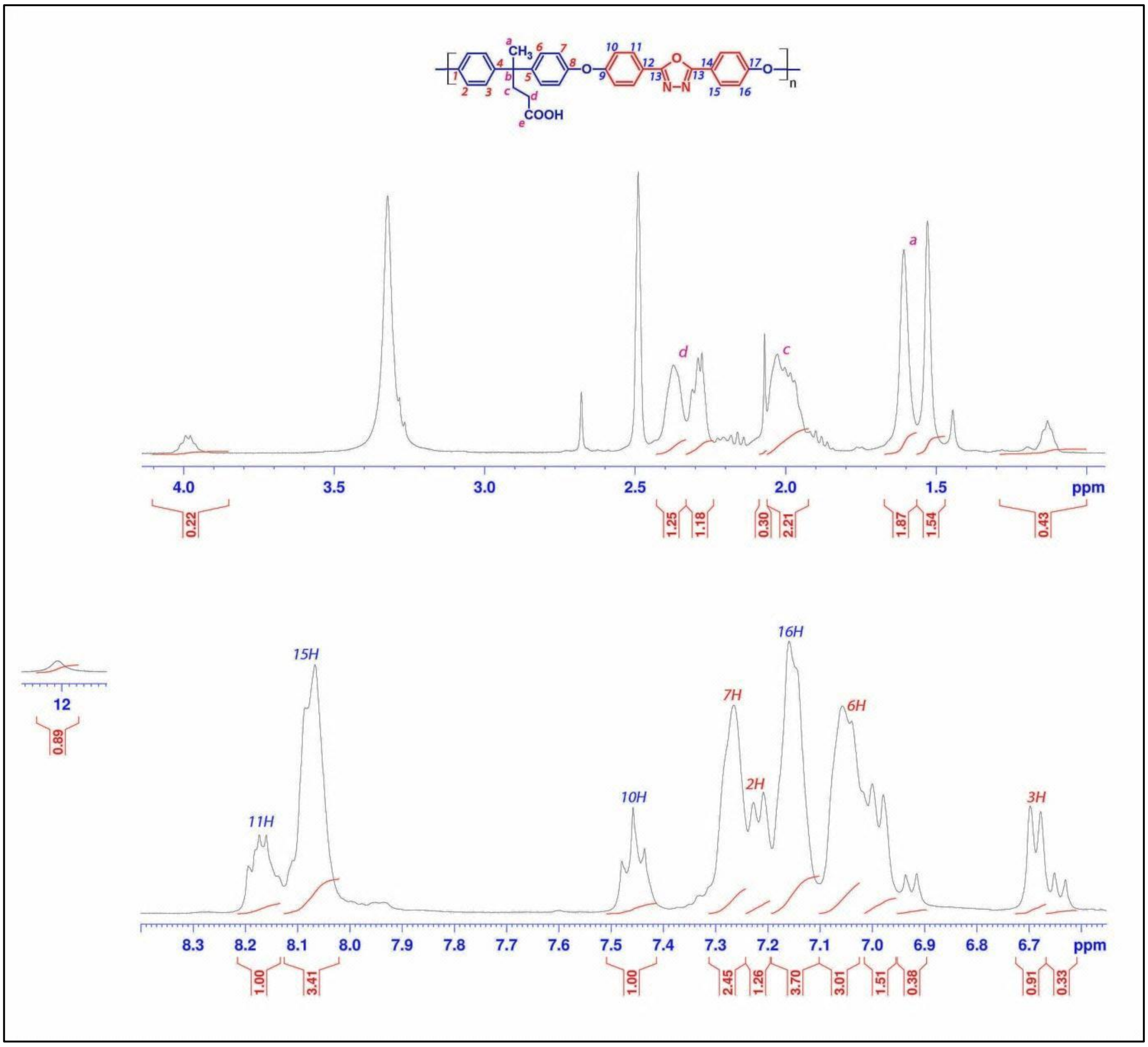


**Figure 1:** $^1$H NMR (DMSO-$d_6$) spectrum of VALPOx Polymer

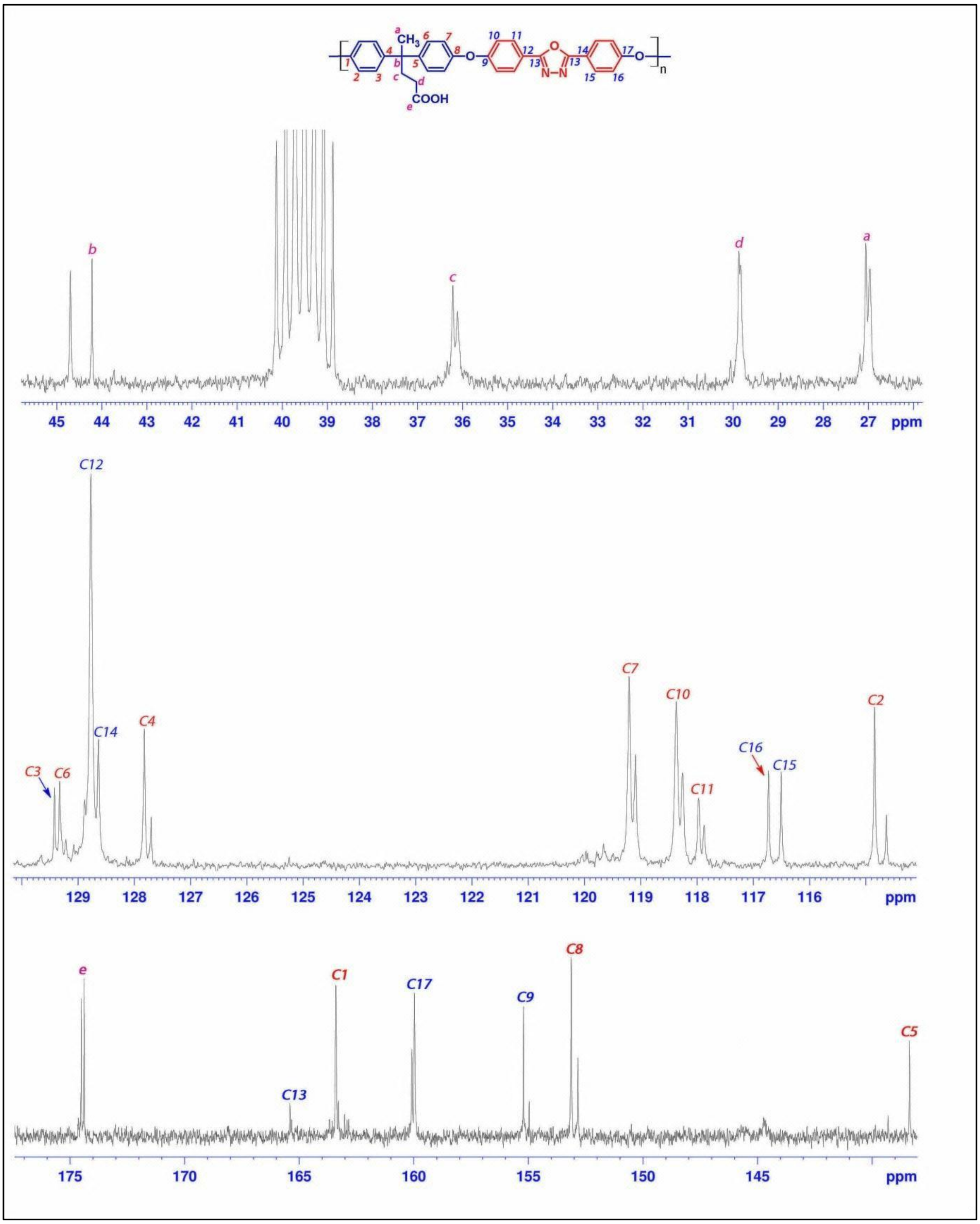

**Figure 2:** $^{13}$C NMR (DMSO-$d_6$) spectrum of VALPOx Polymer

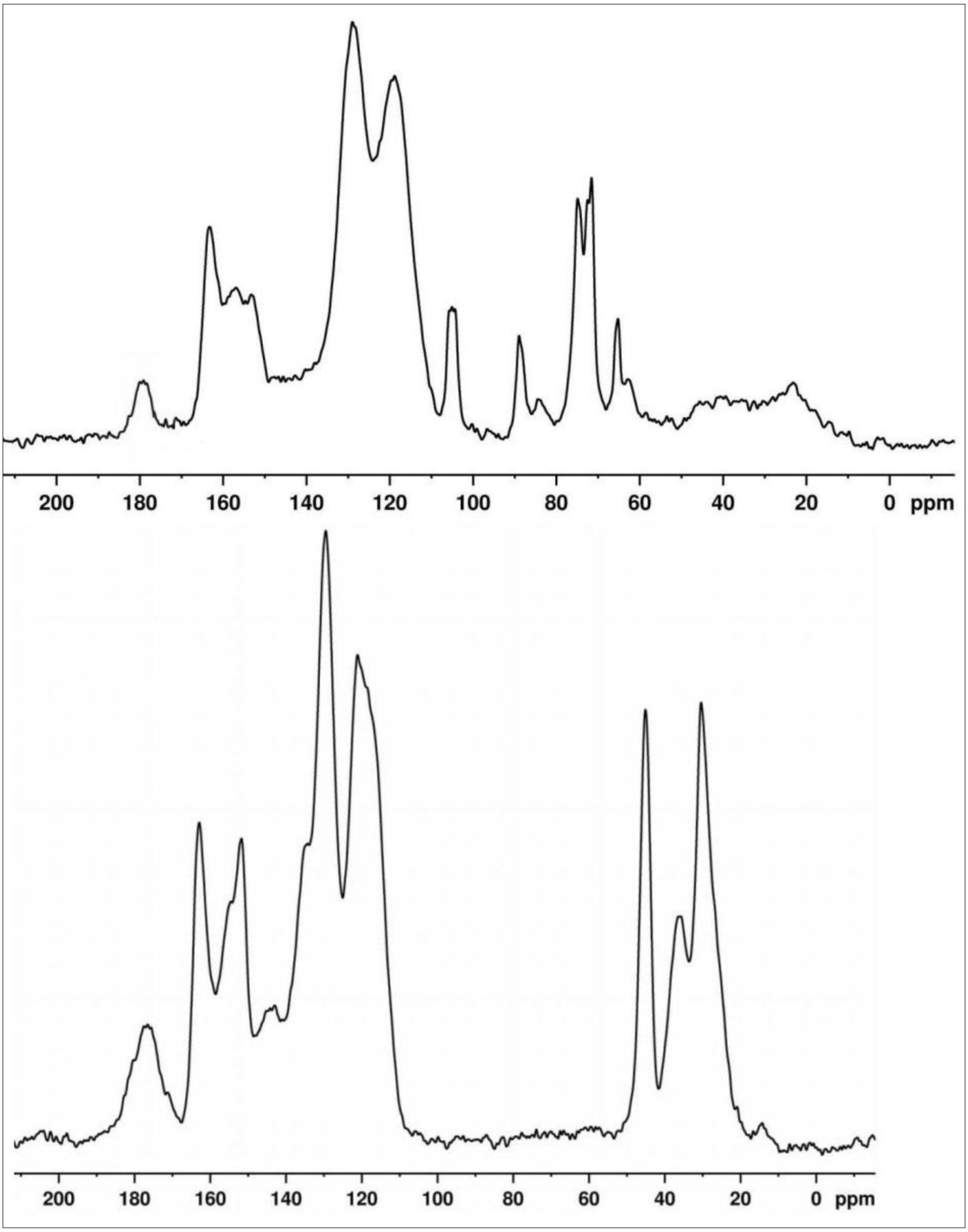


**Figure 3:** Solid state $^{13}C$ spectrum of (a) VALPOx-B-30 (Top) (b) VALPOx Polymer (Bottom)
